# Crystal structure, magneto-structural correlation, thermal and electrical studies of an imidazolium halometallate molten salt: (trimim)[FeCl_4_]†

**DOI:** 10.1039/d0ra00245c

**Published:** 2020-03-23

**Authors:** Palmerina González-Izquierdo, Oscar Fabelo, Garikoitz Beobide, Israel Cano, Idoia Ruiz de Larramendi, Oriol Vallcorba, Jesús Rodríguez Fernández, María Teresa Fernández-Díaz, Imanol de Pedro

**Affiliations:** CITIMAC, Facultad de Ciencias, Universidad de Cantabria 39005 Santander Spain depedrovm@unican.es; Institut Laue-Langevin BP 156X, F-38042 Grenoble Cedex France gonzalez-izquierdo@ill.fr fabelo@ill.fr; Departamento de Química Inorgánica, Facultad de Ciencia y Tecnología, Universidad del País Vasco Apartado 644, E-48080 Bilbao Spain garikoitz.beobide@ehu.es; School of Chemistry, University of Nottingham NG7 2RD Nottingham UK; ALBA Synchrotron Light Source Cerdanyola del Vallés Barcelona Spain

## Abstract

A novel imidazolium halometallate molten salt with formula (trimim)[FeCl_4_] (trimim: 1,2,3-trimethylimidazolium) was synthetized and studied with structural and physico-chemical characterization. Variable-temperature synchrotron X-ray powder diffraction (SXPD) from 100 to 400 K revealed two structural transitions at 200 and 300 K. Three different crystal structures were determined combining single crystal X-ray diffraction (SCXD), neutron powder diffraction (NPD), and SXPD. From 100 to 200 K, the compound exhibits a monoclinic crystal structure with space group *P*2_1_/*c*. At 200 K, the former crystal system and space group are retained, but a disorder in the organic cations is introduced. Above 300 K, the structure transits to the orthorhombic space group *Pbcn*, retaining the crystallinity up to 400 K. The study of the thermal expansion process in this temperature range showed anisotropically evolving cell parameters with an axial negative thermal expansion. Such an induction occurs immediately after the crystal phase transition due to the translational and reorientational dynamic displacements of the imidazolium cation within the crystal building. Electrochemical impedance spectroscopy (EIS) demonstrated that this motion implies a high and stable solid-state ionic conduction (range from 4 × 10^−6^ S cm^−1^ at room temperature to 5.5 × 10^−5^ S cm^−1^ at 400 K). In addition, magnetization and heat capacity measurements proved the presence of a three-dimensional antiferromagnetic ordering below 3 K. The magnetic structure, determined by neutron powder diffraction, corresponds to ferromagnetic chains along the *a*-axis, which are antiferromagnetically coupled to the nearest neighboring chains through an intricate network of superexchange pathways, in agreement with the magnetometry measurements.

## Abstract 2

We present a novel halometallate molten salt based on imidazolium cation with two structural transitions from 100 to 400 K which has been studied by X-ray and neutron diffraction techniques. Furthermore, the magnetic structure at low temperature and the ionic conductivity is also described.

## Graphical abstract

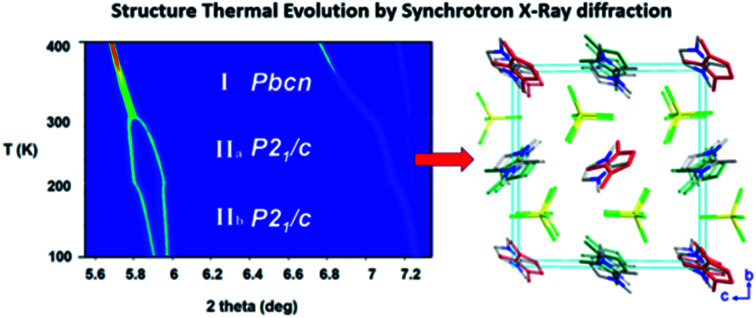

## Introduction

Nowadays, hybrid organic–inorganic materials play a major role in the development of advanced functional materials. The potential combination of the properties of both organic and inorganic components has recently attracted an increasing interest by chemists, physicists, biologists and material scientists.^1–3^ In particular, halometallate complexes exhibit interesting thermal, ferromagnetic, ferroelectric and ferroelastic properties.^4–8^ From a structural point of view, these compounds have been found to exhibit several phase transitions, due to the dynamical reorientation of their organic alkyl moieties.^9–11^ Consequently, this family displays very exciting properties in terms of stability, magnetism and ability of tuning with a wide range of organic and inorganic compounds.

Among them, tetrahaloferrate(iii) complexes are particularly attractive due to their magnetic and crystal behavior.^12,13^ Specifically, imidazolium-based tetrahaloferrate(iii) compounds present long-range magnetic order at low temperature^14–17^ which lead to a highly organized 3-D structure in their condensed phase. Different types of interactions build up their crystal structure, from nonspecific isotropic forces, weak (like van der Waals, solvophobic and dispersion forces) and strong (coulombic), to specific anisotropic forces (such as hydrogen bonding, halogen bonding, dipole–dipole, magnetic dipole and electron pair donor/acceptor interactions). Some of these interactions are strongly influenced by the temperature, while others are not affected due to the anisotropy of both the unit cell and the H-bonding network. As a result, these materials exhibit unusual anisotropic thermal expansion processes, which can be related to the ability of the organic moiety to present translational and reorientational phenomena that govern properties such as magnetism and solid-state ionic conduction. Moreover, from a technological point of view, due to their very interesting features in terms of Lewis-acidity, they are recently being applied as catalysts in reactions such as glycosidation,^18,19^ Michael addition,^20^ desulfurization^21^ and CO_2_ fixation into cyclic carbonates,^22^ among others.

Herein we report the synthesis, crystal characterization, ionic conductivity and magnetic properties of a new tetrahaloferrate molten salt containing the 1,2,3-trimethylimidazolium cation and tetrachloridoferrate anion, (trimim)[FeCl_4_]. We have elucidated the three different crystal structures of this compound in the solid state by single crystal X-ray diffraction (SCXD), neutron powder diffraction (NPD), and variable-temperature synchrotron X-ray powder diffraction (SXPD), which are determined by the effect of the molecular thermal motion of this unexplored cation. In addition, we have carried out a profound study of the magneto–structural correlations.

## Experimental section

### Synthesis

Anhydrous FeCl_3_ (99.9%, Aldrich), 1,2-dimethylimidazole (98%, Sigma-Aldrich), acetonitrile (HPLC grade, Vetec), methyl iodide (98%, Acros) and other chemicals were purchased from commercial sources and used without any further purification. All manipulations were performed in air atmosphere, except otherwise stated in the text.

#### Synthesis of 1,2,3-trimethylimidazolium iodide (trimim)I

1,2-dimethylimidazole (9.52 g, 98 mmol) was dissolved in acetonitrile (150 mL), and methyl iodide (10.68 g, 104.12 mmol) was added dropwise to this stirred solution cooled in an ice bath. Thereafter, the reaction mixture was refluxed at 82 °C for 24 h. The reaction was cooled down and the solvent was removed under vacuum. The remaining solid was re-crystallized in a mixture of acetone: acetonitrile, yielding a white solid (14.60 g, 71% yield). IR: *ν* (cm^−1^) 3435, 3366, 3103, 2106, 1627, 1588, 1412, 1250, 1131, 770. ^1^H NMR (400 MHz, D_2_O) *δ* 7.32 (s, 2H, N–C*H*–C*H*–N), 3.79 (s, 6H, N–C*H*_3_), 2.60 (s, 3H, CH_3_). ^13^C{^1^H} NMR (101 MHz, D_2_O) *δ* 144.7 (s, C_q_, N–*C*–N), 121.8 (s, N–*C*H–*C*H–N), 34.7 (s, N–*C*H_3_), 9.0 (s, *C*H_3_). ESI-HRMS *m*/*z*: [M]^+^ calcd for C_6_H_11_N_2_ 111.0917; found 111.0927. [M]^−^ calcd for I 126.9050; found 126.9402 (Fig. S1–S3 of ESI†).

#### Synthesis of 1,2,3-trimethylimidazolium chloride (trimim)Cl

(trimim)I (5 g, 21 mmol) was dissolved in distilled water (500 mL) and passed through an anion exchange Amberlite IRA-400 column (OH^−^ form) to yield an iodide free solution of 1,2,3-trimethylimidazolium hydroxide (test by the Volhard method). The hydroxide solution was neutralized with hydrochloric acid (36%, Vetec), and water was removed under vacuum at 100 °C with a rotary evaporator. Then, the solid product was dissolved in dichloromethane and dried over anhydrous Na_2_CO_3_. Finally, the carbonate was filtered off and the solvent was removed under vacuum affording a white solid (4.80 g, 92% yield). IR: *ν* (cm^−1^) 3425, 3371, 3101, 2105, 1625, 1589, 1410, 1248, 1127, 768. ^1^H NMR (400 MHz, D_2_O) *δ* 7.30 (s, 2H, N–C*H*–C*H*–N), 3.77 (s, 6H, N–C*H*_3_), 2.57 (s, 3H, CH_3_). ^13^C{^1^H} NMR (101 MHz, D_2_O) *δ* 144.7 (s, C_q_, N–*C*–N), 121.7 (s, N–*C*H–*C*H–N), 34.5 (s, N–*C*H_3_), 8.6 (s, *C*H_3_). ESI-HRMS *m*/*z*: [M]^+^ calcd for C_6_H_11_N_2_ 111.0917; found 111.0927 (Fig. S4–S6 of ESI†).

#### 1,2,3-Trimethylimidazolium tetrachloroferrate (trimim)FeCl_4_

A solution of (trimim)Cl (0.953 g, 6.5 mmol) in methanol (10 mL) was mixed with a solution of anhydrous FeCl_3_ (1.054 g, 6.5 mmol) in methanol (10 mL). The mixture reacted instantaneously and the desired (trimim)FeCl_4_ was obtained as a yellow solid product (1.999 g, 100% yield). Finally, single-crystals suitable for X-ray diffraction were grown by recrystallization of this compound in a mixture 2-propanol : 1-heptanol 1 : 1.

### Physical measurements of (trimim)[FeCl_4_]

Details of SCXRD, SXPD, magnetic and heat capacity measurements, EIS, NPD measurements are thoroughly described in the ESI.†

## Results and discussions

### Thermal analysis, variable-temperature synchrotron and neutron powder diffraction

The title compound transits to the liquid state at a considerably higher temperature (192 °C; see DSC analysis in Fig. S7, ESI†) than other tetrachloridoferrate complexes with less symmetric imidazolium cations, since the melting point depends on the symmetry of the organic cation, among other factors (see Table S1, ESI†).^14–17^ For instance, (emim)[FeCl_4_],^14^ (emim = 1-ethyl-3-methylimidazolium) is liquid at room temperature (melting point = 12 °C). Furthermore, a less electronegative halide ion (Br instead of Cl) rises the melting point; From 5 °C in the (dimim)[FeX_4_],^15,23^ to 35 °C in (edimim)[FeX_4_]^24^ (X = Cl and Br); (dimim = 1,3-dimethylimidaolium) and (edimim = 1-ethyl-2,3-dimethylimidaolium).

**Fig. 1:**
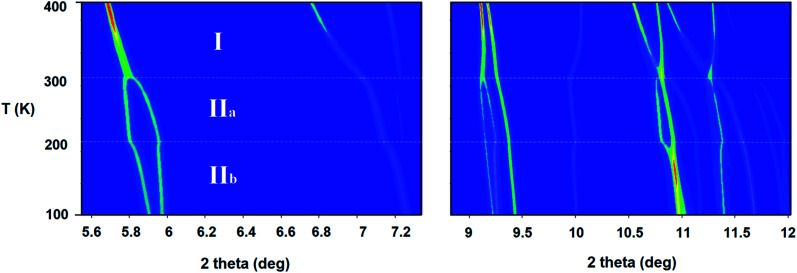
2D contours of the temperature dependence of the selected 2*θ* regions of SXPD patterns between 100 and 400 K of (trimim)[FeCl_4_]. The dashed lines represent the thermo-structural transitions near 200 and 300 K.

SXPD from MSPD beamline (BL04) at ALBA synchrotron (*λ* = 0.62 Å) was employed to study in detail the thermal expansion process and structural transitions of (trimim)[FeCl_4_] from 100 to 400 K under ambient-pressure conditions. The powder patterns exhibit two thermo-structural variations near 200 and 300 K (see Fig. 1), in good agreement with those obtained from DSC. These transitions are related to a change in the molecular conformation of the crystal structure and are associated to the ionic mobility of the imidazolium cation,^25,26^ as it will be discussed below and in the electrochemical impedance spectroscopy (EIS) section.

On one hand, phase II_b_ (located at 100–200 K) was solved by single crystal X-ray diffraction at 150 K. The crystallographic information and refinement parameters are listed in ESI (Table S2†). At this temperature, the complex crystallizes in the monoclinic system, space group *P*2_1_/*c*, with *a* = 6.541(5), *b* = 14.158(5), *c* = 13.351(5) Å, and *β* = 95.590(5)°. On the other hand, phases II_a_ and I were solved by SXPD at 250 and 400 K, respectively. The intermediate phase II_a_ exhibits the same crystal system and space group as the phase II_b_, but promotes a disorder in the organic cations due the increase in the thermal agitation (cell parameters at 250 K: *a* = 6.62(11), *b* = 14.2(2), *c* = 13.6(2) Å and *β* = 95.90(12)°). This disorder above 200 K was confirmed by synchrotron and neutron diffraction measurements (see Fig. S8, ESI†), and by the specific heat curves discussed below. The compound transits to an orthorhombic phase with the higher symmetry space group *Pbcn* above 300 K (phase I). It is worth noting that the organic counterions are also disordered in two different positions in this phase. The cell parameters at 400 K are *a* = 10.4559(1), *b* = 9.2217(1) and *c* = 14.0803(2) Å. The supramolecular structure of the three phases consists of alternate layers of cations and anions, as was observed in similar halometallate compounds.^14–17,27,28^

**Fig. 2:**
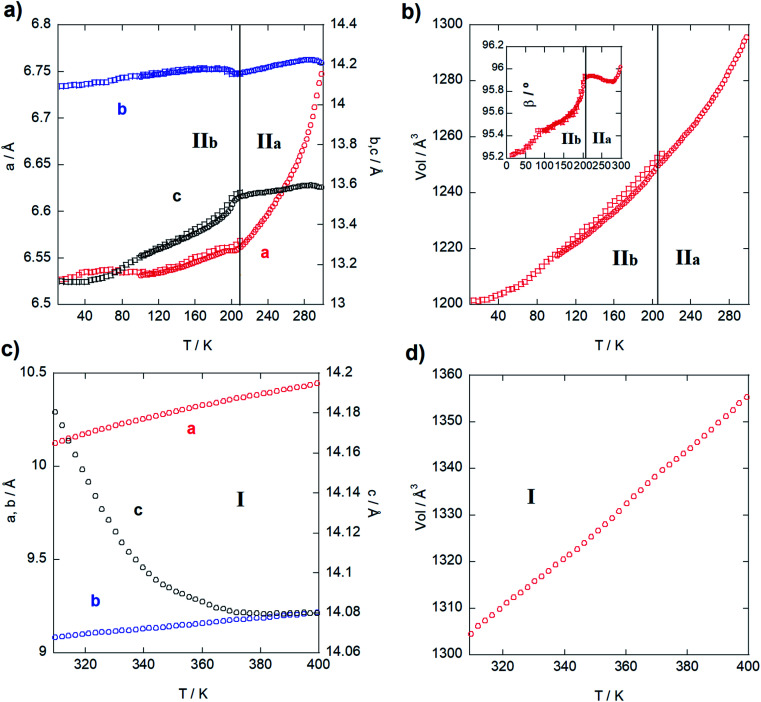
Thermal evolution of *a*- (red), *b*- (blue), *c*- (black) parameters, cell volume and *β* (inset) obtained from the SXPD (circles) and NPD (squares) data for the monoclinic structure from 20 to 300 K (a) and (b) and for the orthorhombic phase from 300 to 400 K (c) and (d).

Next, the thermal evolution of the cell parameters was investigated through a pattern matching analysis of NPD (D1B) and SXPD data from 20 to 300 K and from 300 to 400 K using the FullProf Suite.^29^Fig. 2 gathers the resulting analyses divided in two stages: phases II_b_ and II_a_ (Fig. 2a and b), and phase I (Fig. 2c and d). Firstly, the thermal expansion parameters are positive and anisotropic from 20 to 300 K (monoclinic phases), with the *a*, *b* and *c* parameters growing 0.25, 0.09 and 0.50 Å, respectively (*i.e.* an increase of 3.7, 0.6 and 3.8%). Moreover, the *β* angle grows *ca*. 0.93° (1%). The step observed within the monoclinic range (Fig. 2a) marks a trend change, in agreement with the presence of the aforementioned transition from phase II_b_ to phase II_a_ (*ca.* 200 K). It is noteworthy that *b* and *c* parameters and *β* angle (inset of Fig. 2b) remain very stable, while *a*-parameter increases strongly. Such anisotropic behavior can be ascribed to reorientational dynamical displacements of the imidazolium ring during the heating process (see DSC analysis in Fig. S8, ESI†), as is proved by the analysis of the crystal structure at 150 and 250 K (Fig. 4). This type of dynamical process was also described for other imidazolium salts.^16,17,26,30,31^ Next, *a* and *b* parameters in the orthorhombic phase (from 300 to 400 K; Fig. 2c) increase monotonically 0.33 and 0.13 Å, respectively, while the *c*-axis exhibits a striking negative thermal expansion, decreasing by 0.1 Å (Fig. 2c). Despite this anisotropic evolution of the lattice, the volume increases regularly in both structural phases (Fig. 2b and d) with growth rates of *ca*. 0.35 Å^3^ K^−1^ in the monoclinic phase and 0.52 Å^3^ K^−1^ in the orthorhombic phase.

### Crystal structure of phase I

**Fig. 3:**
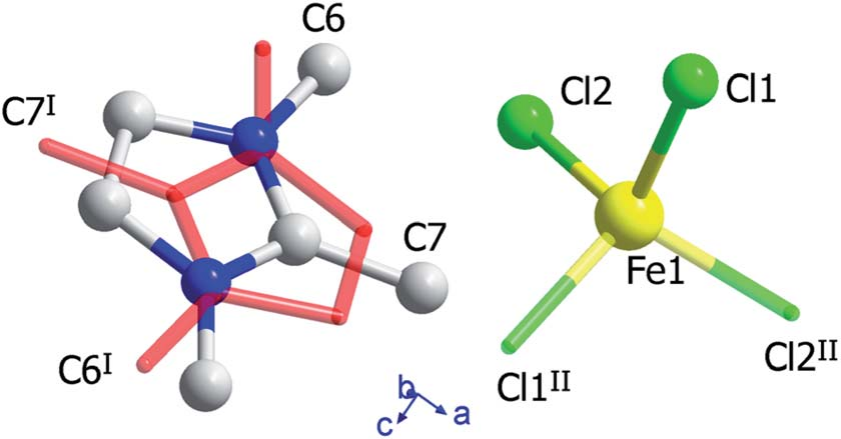
View of the asymmetric unit obtained at 400 K, together with the organic cation and the chloride atoms generated by symmetry operations, represented by sticks (I: 1 − *x*, −*y*, 1 − *z*; II: 2 − *x*, *y*, 1/2 − *z*).

**Fig. 4:**
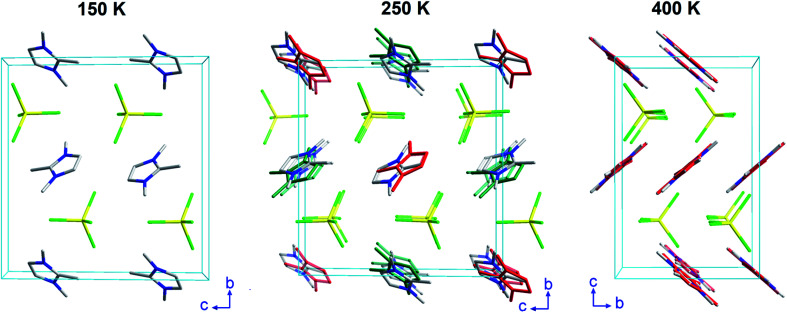
Crystal structure of (trimim)[FeCl_4_] at 150 and 250 K (S.G. *P*2_1_/*c*) along the *c*-axis, and at 400 K (S.G. *Pbcn*) along the *a*-axis. For sake of clarity, the hydrogen atoms belonging to the (trimim)^+^ counterion have been omitted. In addition, a two-color representation has been used for the organic molecules to denote the partial occupation due to the disorder.

The crystal structure of (trimim)FeCl_4_ at 400 K was determined by SXPD due to the low quality of the SCXRD data at this temperature. The obtained atomic positions and Debye Waller factors are gathered in the corresponding CIF file and the Rietveld refinement is shown in Fig. S9c of ESI.†Fig. 3 and 4 illustrate the asymmetric unit and a view of phase I, respectively. Phase I crystallizes in the orthorhombic crystal system, space group *Pbcn*, with *a* = 10.4559(1), *b* = 9.2217(1) and *c* = 14.0803(2) Å. The asymmetric unit is comprised by one [FeCl_4_]^−^ anion and one cation to achieve the charge neutrality. This (trimim)^+^ cation is disordered in two positions related by an inversion symmetry operator (I: 1 − *x*, −*y*, 1 − *z*; Fig. 3) and the disorder was modeled by fixing the occupational factor to 0.5 for each part in the final refinement cycle. This structural disorder arises from the weak bonding network that builds up the supramolecular structure, as was previously reported for similar compounds.^6,14–17,32–34^ The [FeCl_4_]^−^ units shows a tetrahedral geometry in which two chloride atoms are related by a plane of symmetry (II: 2 − *x*, *y*, 1/2 − *z*). This tetrahedron has a mean Cl–Fe–Cl bond angle of 109.5(8)° and an average Fe–Cl bond distance of 2.17(2) Å. In the case of (trimim)^+^ unit, all the refined values for C–C and C–N bond lengths lay in the expected range of other imidazolium-based compounds.^35^

The 3-D assembly can be described as a stacking of organic (trimim)^+^ and inorganic [FeCl_4_]^−^ layers along the crystallographic *c*-axis (Fig. 4). Each of these layers changes of orientation following a A/B/C/D′ stacking sequence (with A/C and B/D corresponding to inorganic and organic layers, respectively). The intralayer Fe⋯Fe distance between neighboring halometallate complexes is *ca*. 7 Å, while the interlayer Fe⋯Fe distances are markedly lengthened due to the intercalation of the cationic layers (shortest values: 8.0–8.8 Å). The crystal packing is sustained by an intricate network of electrostatic and non-covalent interactions.

### Crystal structure of phase II_a_

**Fig. 5:**
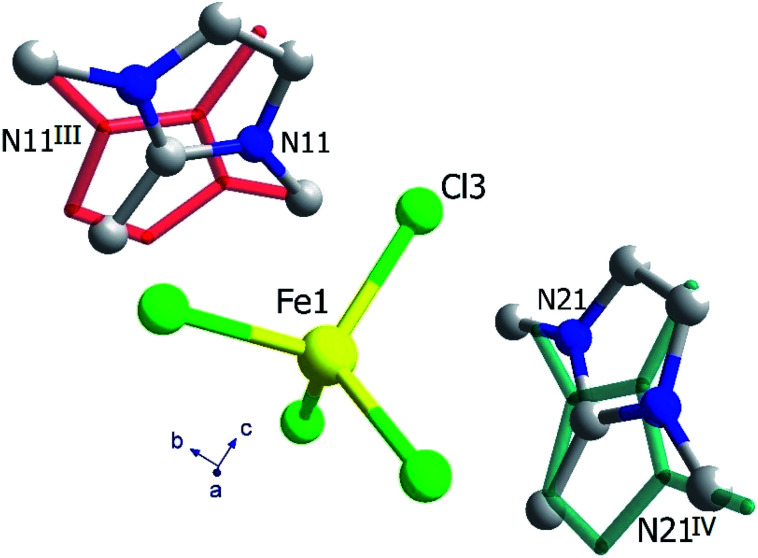
Asymmetric unit of (trimim)[FeCl_4_] at 250 K. The disorder of each of the two symmetrically independent cations is depicted by the overlap on stick representation (III: 2 − *x*, 1 − *y*, 1 − *z*; IV: 2 − *x*, −*y*, 1 − *z*).

The crystal structure at 250 K was also determined by SXPD. The atomic positions and the isotropic Debye–Waller factors were refined through a multi-pattern Rietveld analysis of SXPD and NPD (D1B and D2B beamlines) data (Fig. S9 and S10, ESI†). The resulting atomic coordinates and thermal parameters are listed in the corresponding CIF file at 250 K. The unit cell is shown in Fig. 4 and the asymmetric unit is represented in Fig. 5. The crystal structure in this phase was solved in the monoclinic space group *P*2_1_/*c*, with *a* = 6.6156(1), *b* = 14.2016(2), *c* = 13.5720(2) Å and *β* = 95.9053(9)°. Although there is a group/subgroup relationship between the high temperature phase (*Pbcn*) and the low temperature phase (*P*2_1_/*c*), the refined crystal structures cannot be transformed applying symmetry operators.

The asymmetric unit comprises one [FeCl_4_]^−^ anion and two (trimim)^+^ cations. Each cation is disordered in two positions with equal probability, which are related by an inversion symmetry operator (III: 2 − *x*, 1 − *y*, 1 − *z* and IV: 2 − *x*, −*y*, 1 − *z*, Fig. 5). Unlike the previous case, the anion exhibits four independent positions for the chloride atoms, with a mean Fe–Cl distance of 2.18(5) Å and a Cl–Fe–Cl bond angle of 109(3)°. These values are in agreement with those observed for the previous phase. Regarding the 3-D assembly, the aforementioned stacking sequence of organic and inorganic ions is now along the *c*-axis. Likewise, the Fe⋯Fe lengths agree with those of the orthorhombic phase, for which the shortest intra-layer distance is slightly reduced [6.616(7) Å] and the shortest inter-layer distance is slightly lengthened [8.253(19) Å]. The main distances involved in the bonding network sustaining the crystal packing are listed in Table S3 (ESI†).

### Crystal structure of phase II_b_

This phase was solved by single crystal X-ray diffraction at 150 K. The main crystallographic data and refinement parameters are gathered in Table S2 (ESI†) and the corresponding CIF file. The asymmetric unit consists of one (trimim)^+^ cation and one [FeCl_4_]^−^ anion. The unit cell and the space group are equivalent to the phase II_a_. However, in contrast to II_a_ phase, the organic cations are well ordered at low temperature. Both [FeCl_4_]^−^ and (trimim)^+^ entities comprising the inorganic and organic layers alternate their relative orientation along the *c*-axis following a ABAB sequence (Fig. 4). Phase II_b_ remains down to 1.5 K, as was verified by a multi-pattern Rietveld refinement to SXPD and D2B NPD data at 100 K and to D2B and D1B NPD data at 10 K. The Rietveld refinements at 100 K to the SXPD and NPD data from D2B are shown in Fig. S9 and S11 (ESI†), respectively. In addition, these refinements allowed us to place the hydrogen atoms, and thus study the hydrogen-bonding network at such temperatures (10 and 100 K).

The observed Fe⋯Fe distances are in agreement with those previously reported for other paramagnetic compounds based on halometallate cations.^35–42^

The cation–anion interactions imply an extended H-bonding network and halide–π interactions. Indeed, four hydrogen bonds between the halides and the surrounding (trimim)^+^ cations have been identified at 10 and 100 K, according to IUPAC recommendation for H-bonds (H⋯Cl distance lower than the sum of the vdW radii and the angle greater than 110).^43^ The shortest H-bond takes place between C4–H4⋯Cl2, with a hydrogen contact distance of 2.776(8) and 2.794(7) Å and an angle of 162.5(5) and 162.5(5)° at 10 and 100 K, respectively. Regarding the halide–π interactions, the nearest imidazolium centroid⋯Cl contact [3.6764(16) Å and *θ* = 6.67(11)°, where *θ* stands for the angle between the imidazolium ring plane and the plane formed by the three closest chloride atoms] is within the range of accepted values for anion–π interaction (3.65 Å and *θ* = 0 ± 10°).^44^

### Electrochemical impedance spectroscopy (EIS)

**Fig. 6:**
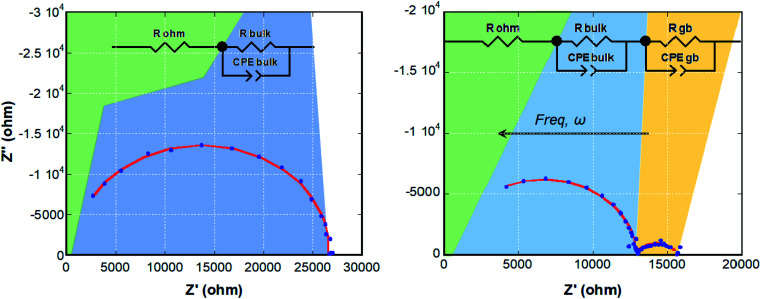
Nyquist diagrams of (trimim)[FeCl_4_] at 330 (left) and 400 K (right). The data fit to the equivalent circuits (insets) is depicted as a solid red line.

The local orientational or rotational disorder of the imidazolium cation in the halometallate molten salt could give rise to a noticeable conductivity as observed in other hybrid organic–inorganic materials that possess aromatic species.^45^ To address this issue, the temperature dependence of the conductivity was assessed by means of electrochemical impedance spectra (Nyquist plots) measured from room temperature (RT) to 400 K in the frequency range 10^−2^ to 10^6^ Hz (selected plots at 330 and 400 K are shown in Fig. 6).

The ESI diagrams were modelled using electric equivalent circuits, in line with the observed physicochemical phenomena. To this end, two parallel connections of a resistor with a Constant Phase Element (CPE) were used in order to simulate the electrochemical processes that take place in the sample (inset in Fig. 6). The Constant Phase Element (CPE), also known as Q-element,^48^ was employed to describe the distribution of relaxation times for the bulk and grain boundary contributions at high and low frequencies, respectively, the latter could be interpreted as an effect attributable to the corrosion of the electrodes, although in this case an irregular signal would be expected. The brick layer model, proposed by Van Dijk and Burggraaf,^46^ was employed to analyze the measurements. The obtained values of capacitance and resistance from these fits are reported in Table S5 (ESI†). The ionic conductivities of the sample can be calculated from the values of resistance through the equation:
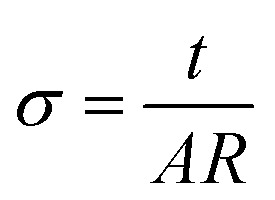
where *t* is the thickness of the measured pellet (cm), *A* is the area of the pellet (cm^2^) and *R* is the resistance obtained from the fit.

Fig. S12 (ESI†) shows the conductivity as a function of temperature. As expected, the conductivity increases with temperature due to the ionic motion. The Arrhenius plots of ionic conductivity of homogeneous electrolytes usually exhibit a straight or curved relationship.^37^ In our case, due to the low statistics of the data set, the fit to these or other more complex models was not performed to avoid parametrization. The conductivity suddenly decreases above 395 K due to the high chloride content, leading to corrosion of the electrodes (Fig. S13, ESI†). Therefore, we were not able to study the conductivity from 395 K to nearly the melting point (465 K). The conductivity values are in the range from 4 × 10^−6^ S cm^−1^ at R.T. to 5.5 × 10^−5^ S cm^−1^ at 400 K. Although these values are lower than those observed for ILs at R.T. in the liquid state (10^−2^ to 10^1^ S cm^−1^).^35,37,47^ The proton conduction in similar imidazolium-based salts has been related to the Grotthus mechanism, taking place through the reorientation of the cation around a preferent axis.^30,49^ This mechanism is in agreement with the structural disorder of (trimim)[FeCl_4_], which shows a local orientational or rotational disorder of the imidazolium cation.

### Magnetic properties

**Fig. 7:**
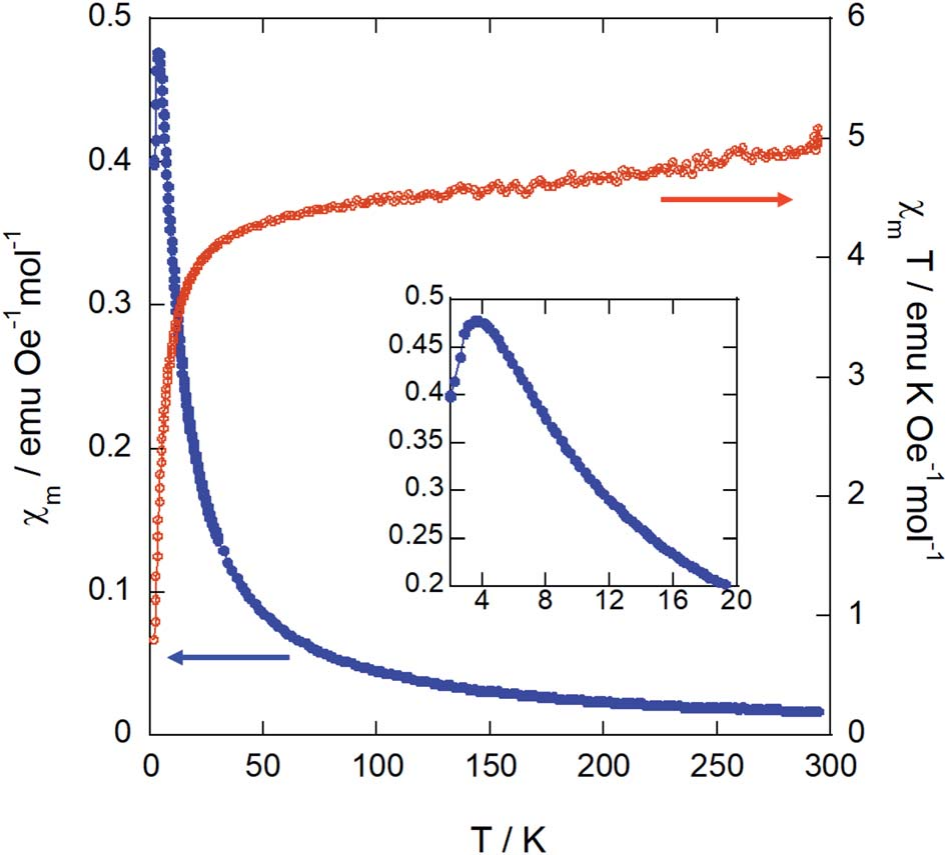
Temperature dependence of *χ*_m_ and *χ*_m_*T* for (trimim)[FeCl_4_] measured at 1 kOe. The inset shows a zoom of the low temperature region of *χ*_m_.

**Fig. 8:**
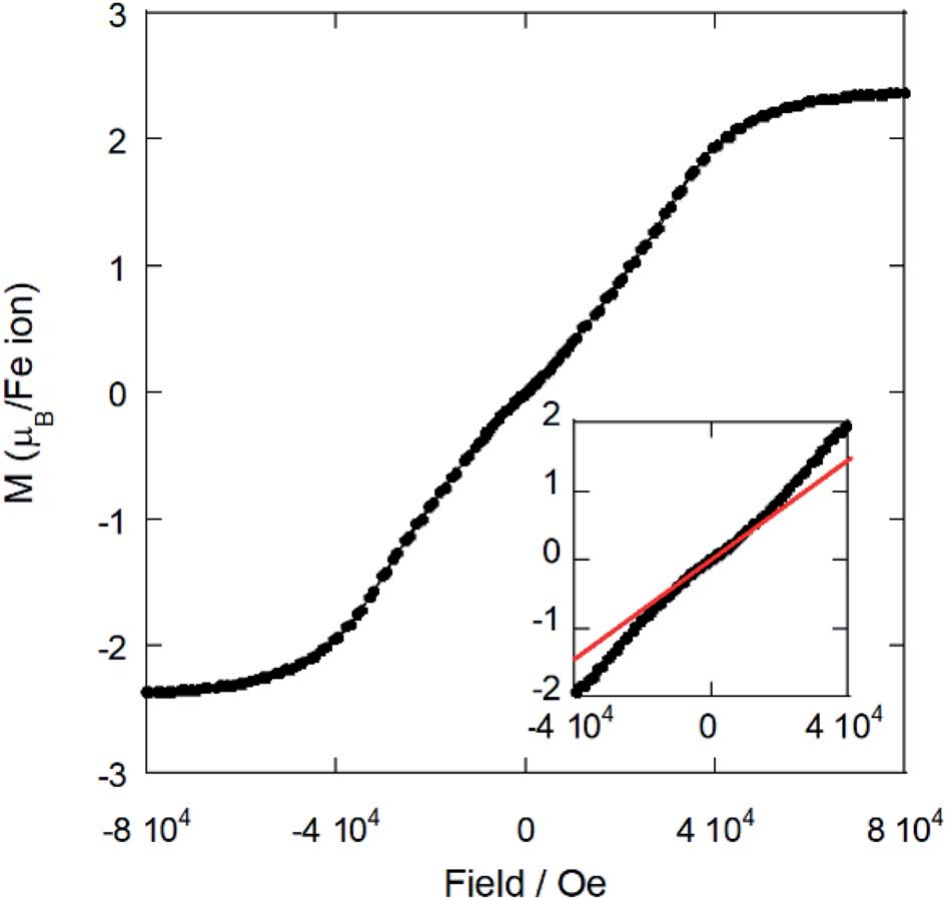
Magnetization *vs.* applied magnetic field at 2 K for (trimim)[FeCl_4_]. The inset displays an enlargement of the low field region. The red line is a guideline to show the possible metamagnetic transition.

Magnetic susceptibility measurements were performed over the temperature range 2–300 K on a powder sample. The plots for the magnetic susceptibility *χ*_m_, *χ*_m_*T vs. T* at 1 kOe and the magnetization *vs.* the applied field are shown in Fig. 7 and 8, respectively.

On one hand, the magnetic susceptibility displays a paramagnetic behavior above 10 K with a *χ*_m_*T* value of 4.82 emu K mol^−1^ Oe^−1^ at R.T., which is slightly higher than the expected value of 4.38 emu K mol^−1^ Oe^−1^ for Fe^3+^ ions with *S* = 5/2 magnetic spin.^48^ The magnetic susceptibility data in the paramagnetic region can be fitted to a Curie–Weiss law (Fig. S13, ESI†) with a Weiss temperature of *θ*_p_ = −6.3 (1) K and an effective paramagnetic moment *μ*_eff_ = 6.22(1) *μ*_B_ per Fe^3+^ ion. This effective paramagnetic moment is also slightly higher than the expected value for high spin d_5_ Fe^3+^ ions (*μ*_eff_ = 5.92 *μ*_B_ per Fe ion), but it is in good agreement with the previously reported data for other paramagnetic compounds based on [FeCl_4_]^−^ ions.^6,15,49^

Note that the fit to the Curie–Weiss law does not follow completely the expected straight line for fully paramagnetic compounds, which indicates the competition between different magnetic interactions. The negative deviation of *χ*_m_*T* with decreasing temperature suggests an antiferromagnetic behavior. Accordingly, the magnetic susceptibility reaches a maximum at 3.8 K and drops when the temperature decreases down to 2 K (see the inset of Fig. 7), confirming the existence of long-range antiferromagnetic order. The 3-D character of this transition was also corroborated by specific heat and neutron diffraction measurements (discussed below).

On the other hand, the field dependence of the magnetization at 2 K (Fig. 8) changes in curvature at *ca*. 5 kOe (inset of Fig. 7), probably due to the presence of a weak metamagnetic component. The value of magnetization at 80 kOe is 2.36 *μ*_B_/Fe^3+^ ion, which is far away from the expected fully-saturated value of 5 *μ*_B_/Fe for a Fe^3+^ ion. This indicates the existence of a non-negligible magnetocrystalline anisotropy in the 3-D magnetic structure, as was previously reported for other halometallate compounds.^15,16^

### Specific heat measurements

**Fig. 9:**
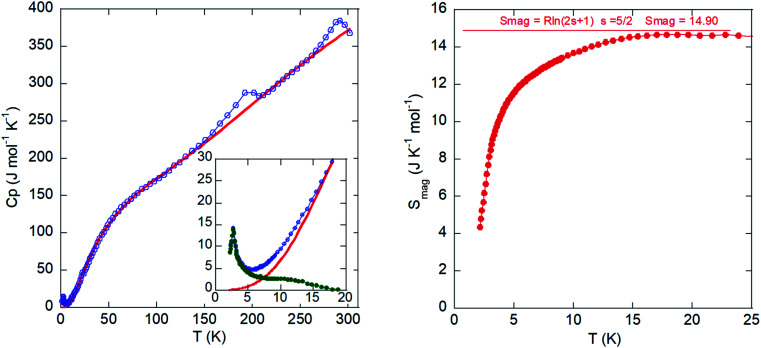
Left: Specific heat (blue circles) as a function of temperature. The solid red line corresponds to the fit to the Debye model. Inset: magnification of the low temperature range. The green markers represent the magnetic contribution. Right: Magnetic entropy as a function of temperature. The solid red line represents the theoretical value for a Fe^3+^ ion with spin 5/2.

The temperature dependence of the molar heat capacity (*C*_P_) is presented in Fig. 9 (left). Calorimetric measurements in the absence of an external magnetic field reveal a maximum (Δ*C*_P_ = 9.9 J mol^−1^ K^−1^) centered at 2.7 K (see lower inset in Fig. 9, left). This anomaly displays the typical λ-shape appearance of a second-order transition, which can be related to the establishment of a 3-D antiferromagnetic order in good agreement with the magnetic susceptibility data. Above the order temperature, *C*_P_ increases continuously as a result of the phonon contribution, with two peaks at 200 and 300 K corresponding to the phase transitions, and does not show any tendency to saturation even up to R.T., where *C*_P_ (375 J mol^−1^ K^−1^) is still far from the expected value according to the Dulong and Petit law (575 J mol^−1^ K^−1^). This behavior is attributed to the presence of many hydrogen atoms within the imidazolium cation, which display very high excitation energies. The phonon contribution (*C*_pho_) must be determined to extract the magnetic contribution (*C*_mag_) from the specific heat data (*C*_p_). An accurate calculation for *C*_pho_ is difficult due to the absence of a suitable non-magnetic isomorphous compound. Thus, we obtained an estimation of *C*_pho_ using the Debye model^50^ and considering three Debye temperatures:^51^ a lower one (*θ*_1_) related to heavy atoms such as Fe and Cl (*n*_1_), an intermediate one (*θ*_2_) linked to C and N atoms (*n*_2_), and a higher one (*θ*_3_) associated with H atoms (*n*_3_). The best fit to the experimental data from 15 to 300 K is obtained for *θ*_1_ =102.7 K, *θ*_2_ =256.4 K, *θ*_3_ = 1399 K, *n*_1_ = 3.7, *n*_2_ = 3.9, and *n*_3_ = 16.4 atoms. The peaks corresponding to the structural phase transitions do not show the typical λ-shape. Therefore, these “sluggish” transitions are difficult to define, as they do not occur instantaneously. We employed the Boltzmann equation to study these transitions, Δ*S* = *R* ln *N*, where Δ*S* corresponds to the area under the curve (*C*_P_ − *C*_pho_)/*T* in the temperature range in which the transition occurs, *R* is the gas constant, and *N* is the ratio of geometrically distinguishable orientations. For the low temperature transition, we obtained *N* = 1.71, which is close to *N* = 2, pointing to a phase transition with two-fold order-disorder character, as we showed previously in the structural section. For the high temperature transition, the value determined for *N* was 1.21. Nevertheless, in this case the disorder in the organic cation is still present but the transition implies a change on the complete crystal structure, which renders this value meaningless as other factors should be taken into account.

The temperature dependence of the magnetic contribution (*C*_mag_) is shown in the inset of Fig. 9 (left). The jump of *C*_mag_ at 2.7 K, Δ*C*_mag_ = 14.3 J K^−1^ mol^−1^, is 27% lower than that expected for an antiferromagnetic structure with *S* = 5/2 (Δ*C*_mag_ = 19.6 J K^−1^ mol^−1^) within the molecular field approximation.^52^ This feature can be attributed to the presence of spin fluctuations.

Finally, the magnetic entropy variation calculated as 
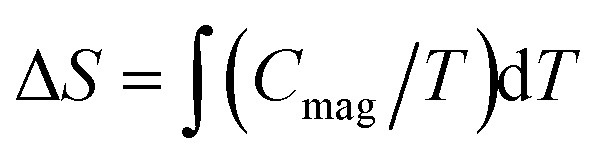
 is shown in Fig. 9 (right). This parameter increases with temperature, reaching a value of *ca*. 14.9 J K^−1^ mol^−1^ around 15 K that corresponds to the theoretical entropy coming from the magnetic specific heat based on the degenerate freedom of *S* = 5/2. This result confirms the presence of a long-range antiferromagnetic order below 2.7 K and a high-spin state for the Fe^3+^ ions (*S* = 5/2).

### Magnetic structure determination

**Fig. 10:**
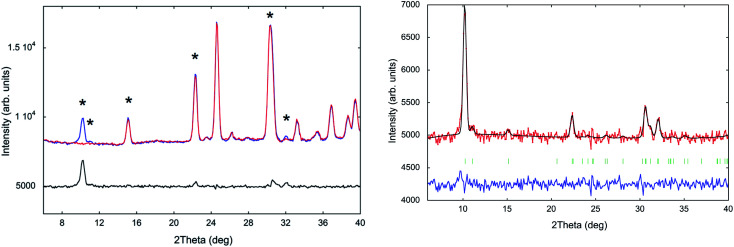
Left: Neutron diffraction profiles of (trimim)[FeCl_4_] at 10 (red) and 1.5 K (blue) and the difference pattern between them (black), obtained in D1B. Right: Observed (red points) and calculated (black solid line) powder difference diffraction pattern of (trimim)[FeCl_4_] obtained in D1B between 1.5 and 10 K. Positions of the Bragg reflections are represented by vertical red bars (magnetic: red; nuclear and magnetic: blue). The observed-calculated difference pattern is depicted as a blue line at the bottom of the figure.

The evolution of neutron diffraction patterns with the temperature in the range 10–220 K (Fig. S7, ESI†) was collected using the high flux D1B diffractometer, working at *λ* = 2.52 Å. A comparative view of a pattern in the paramagnetic state (10 K) and a magnetic ordered one (1.5 K) reveals the presence of a new sharp Bragg peak and magnetic contributions on top of four of the nuclear peaks (marked with asterisks in Fig. 10, left). This is a fingerprint of the existence of 3-D magnetic ordering, in good agreement with the results obtained from the magnetometry and specific heat measurements (Néel temperature; *T*_N_ = 3.8 and 2.7 K, respectively). Due to the weak magnetic signal, the overlap between nuclear reflections and the considerable background, the nuclear contribution has been subtracted from the pattern at 1.5 K (by subtracting the intensity of the pattern at 10 K). This “difference” pattern (Fig. 10 right) isolates the magnetic contribution. Thus, a more accurate indexing of the magnetic reflections and an explanation of the magnetic structure can be undertaken. Both diffraction patterns were normalized to the same monitor in order to obtain the best difference pattern. The validity of this approach was confirmed through a Rietveld refinement in the paramagnetic phase, close to the magnetic phase transition, which shows that the *P*2_1_/*c* space group is maintained. This refinement was performed using the atomic coordinates of the nuclear part obtained from the multipattern Rietveld refinement to the D1B and D2B data at 10 K. The same model was used at 1.5 K, in which the additional elastic intensities can be indexed with a propagation vector *k* = (0, 0, 0), indicating that the magnetic cell corresponds to the nuclear cell.

**Fig. 11:**
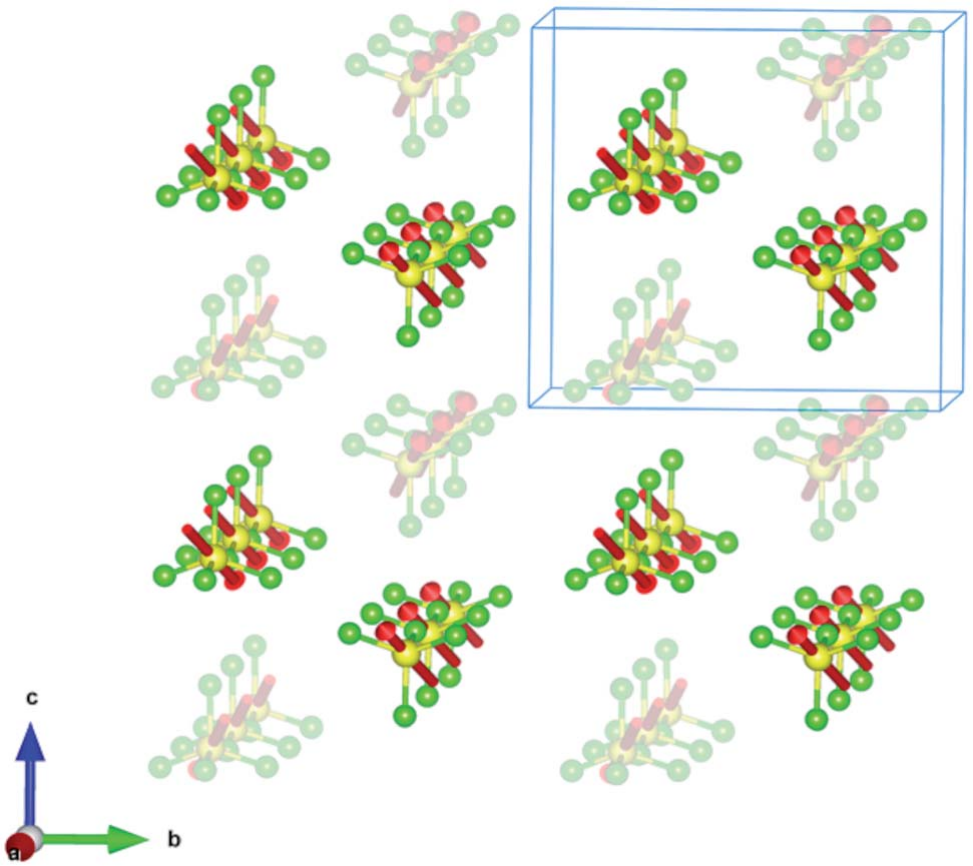
Magnetic structure of (trimim)[FeCl_4_] at 1.5 K. Transparencies have been used to highlight the corrugated antiferromagnetic layers pillared along the c-axis. Color codes: yellow, iron; green, chloride; red, magnetic spin. The organic cations have been omitted for the sake of clarity.

In order to determine the magnetic structure, the irreducible representations (irreps) compatible with the propagation vector were investigated using the Bertaut's symmetry analysis method^53^ implemented in the BasIreps program included in the FullProf suite.^29^ This approach allowed us to determine the symmetry operations applied to the magnetic moments of Fe^3+^ ion within the magnetic unit cell. The total magnetic representation of the propagation vector group can be decomposed in four irreducible representations (for more details see the magnetic model calculation in Table S6, ESI†). The best magnetic model is described by irreducible representation *Γ*_2_, associated to the magnetic space group *P*2_1_/*c* (the fit is shown in Fig. 10, right). This model corresponds to ferromagnetic chains along the *a*-axis, which are antiferromagnetically coupled to the nearest neighbouring chains along the [011] and [0−11] directions, and symmetry related, forming corrugated planes, pillared along the *c*-axis (Fig. 11). This model provide a strictly antiferromagnetic structure (*i.e.* the total magnetization vector per unit cell is null), which is compatible with the macroscopic measurements previously described. The observed total magnetic moment is 3.42(5) *μ*_B_, which is lower than the expected 5 *μ*_B_ for a Fe^3+^ion with *S* = 5/2. This difference can be produced by two main effects. The first one can be attributed to the fact that non-polarized neutrons are only sensitive to the magnetic moments onto the magnetic atoms. Thus, the contribution of surrounding chlorine atoms to the net magnetic moment (caused by a significant spin density delocalization) is not measured as observed in similar compounds.^15^ The second effect can be ascribed to a system that has not reached a complete ordering because the measurements are performed very close to the magnetic transition, according to the specific heat analyses (2.7 K).

## Conclusions

In summary, a new halometallate molten salt based on imidazolium cation, (trimim)[FeCl_4_], was synthetized and fully characterized. The crystal structure is sustained by an intricate network of non-covalent interactions (hydrogen bonding, anion−π, and), which are affected in different ways by temperature. The local orientational or rotational dynamic disorder of the imidazolium cation during the heating promotes an anisotropic thermal expansion and crystal phase transition from monoclinic (*P*2_1_/*c*) to orthorhombic phase (*Pbcn*) at *ca*. R.T. Moreover, this fact also favors a high and stable solid-state ionic conduction.

The supramolecular network yields a three dimensional magnetic ordering below 3 K, confirmed by a combination of specific heat measurements and neutron powder diffraction. Furthermore, it was found that the magnetic structure was strictly antiferromagnetic determined by neutron diffraction, which is in a good agreement with the macroscopic magnetometry measurements. Indeed, based on the obtained magnetic structure, a competition between ferro- and antiferromagnetic interactions is observed, with a final predominance of the antiferromagnetic behavior.

## Conflicts of interest

There are no conflicts of interest to declare.

## Supplementary Material

RA-010-D0RA00245C-s001

RA-010-D0RA00245C-s002
