# Correction: Crystal structure, magneto-structural correlation, thermal and electrical studies of an imidazolium halometallate molten salt: (trimim)[FeCl_4_]

**DOI:** 10.1039/d0ra90044c

**Published:** 2020-04-29

**Authors:** Palmerina González-Izquierdo, Oscar Fabelo, Garikoitz Beobide, Israel Cano, Idoia Ruiz de Larramendi, Oriol Vallcorba, Jesús Rodríguez Fernández, María Teresa Fernández-Díaz, Imanol de Pedro

**Affiliations:** CITIMAC, Facultad de Ciencias, Universidad de Cantabria 39005 Santander Spain depedrovm@unican.es; Institut Laue-Langevin BP 156X F-38042 Grenoble Cedex France gonzalez-izquierdo@ill.fr fabelo@ill.fr; Departamento de Química Inorgánica, Facultad de Ciencia y Tecnología, Universidad del País Vasco Apartado 644 E-48080 Bilbao Spain garikoitz.beobide@ehu.es; School of Chemistry, University of Nottingham NG7 2RD Nottingham UK; ALBA Synchrotron Light Source Cerdanyola del Vallés Barcelona Spain

## Abstract

Correction for ‘Crystal structure, magneto-structural correlation, thermal and electrical studies of an imidazolium halometallate molten salt: (trimim)[FeCl_4_]’ by Palmerina González-Izquierdo *et al.*, *RSC Adv.*, 2020, **10**, 11200–11209. DOI: 10.1039/D0RA00245C.

The authors regret the omission of a funding acknowledgement in the original article. This acknowledgement is given below.

The Authors would like to acknowledge support from FILL2030, a European Union project within the European Commission’s Horizon 2020 Research and Innovation programme under grant agreement no. 731096.

The Royal Society of Chemistry apologises for these errors and any consequent inconvenience to authors and readers.

## Supplementary Material

