# Supplementary material for: Crystal structure, magneto-structural correlation, thermal and electrical studies of an imidazolium halometallate molten salt: (trimim)[FeCl4]
Source: RSC Adv. 2020 Mar 23;10(19):11200–9. doi: 10.1039/d0ra00245c (PMC9050550; doi:10.1039/d0ra00245c)
Supplement: RA-010-D0RA00245C-s001 [file RA-010-D0RA00245C-s001.pdf]

Electronic Supplementary Material (ESI) for RSC Advances.
This journal is © The Royal Society of Chemistry 2020

ELECTRONIC SUPPORTING INFORMATION

Crystal Structure, Magneto-Structural Correlation, Thermal and Electrical Studies of an Imidazolium Halometallate Molten Salt: (trimim)[$FeCl_4$]

Palmerina González-Izquierdo,[a,b] Oscar Fabelo,[b] Garikoitz Beobide,[c] Israel Cano,[d] Idoia Ruiz de Larramendi,[c] Oriol Vallcorba,[e] Jesús Rodríguez Fernández,[a] María Teresa Fernández-Díaz[b] and Imanol de Pedro[a]

[a] CITIMAC, Facultad de Ciencias, Universidad de Cantabria, 39005 Santander.

[b] Institut Laue-Langevin, BP 156X, F-38042 Grenoble Cedex, France.

[c] Departamento de Química Inorgánica, Facultad de Ciencia y Tecnología, Universidad del País Vasco, Apartado 644, E-48080, Bilbao, Spain.

[d] School of Chemistry, University of Nottingham, NG7 2RD, Nottingham, UK.

[e] ALBA Synchrotron Light Source, Cerdanyola del Vallés, Barcelona, Spain.

Contents

1. **Experimental procedures…………………………………………………..… S2**
2. **Characterization of (trimim)I and (trimim)Cl……….............................. S4**
3. **DSC analysis…………………………………………….......................... S7**
4. **Variable temperature NPD………………………………......................... S7**
5. **SXPD and NPD (D2B) diffraction analysis ……………….…………….. S8**
6. **Ionic conductivity as a function of temperature……………………….. S10**
7. **Curie-Weiss fitting………………………...……………………………… S10**
8. **Tables…………………………………………………………………......................... S11**
9. **Details of magnetic model calculations……………………………………. S12**
10. **References………………………………………………………………… S13**

1. Experimental procedures

Nuclear Magnetic Resonance (NMR)
^{1}H and ^{13}C NMR spectra were recorded on a Bruker DPX400 400 MHz nuclear magnetic resonance spectrometer.

Electrospray Ionization Mass spectrometry (ESI-MS)
ESI-MS analyses were carried out on a Bruker ESI-TOF MicroTOF II.

Fourier Transform Infrared Spectroscopy (ATR FT-IR)
ATR FT-IR measurements were performed on a Bruker Alpha Series FT-IR spectrometer equipped with an attenuated total reflectance (ATR) module. The ATR FT-IR spectra were recorded by collecting 24 scans of a sample in the ATR module.

Thermal analysis
A Setaram calorimeter (DSC131) was used for the thermal analyses from 20 to 250 ºC under nitrogen atmosphere at a heating rate of 10 K/min. For this experiment, *ca*. 5 mg of sample were used, and blank runs were performed.

Single-crystal X-ray diffraction and structure determination:

The crystal structure of (trimim)[$FeCl_4$] was determined by single crystal X-ray diffraction at 150 K. Data were collected using Mo-Kα radiation (0.71073 Å) in an Agilent Technologies Supernova diffractometer. A single crystal of the compound with approximate dimensions 0.11 mm × 0.19 mm × 0.23 mm was employed. The data reduction was performed with the CrysAlis PRO program.[1] Data were corrected for Lorentz and polarization effects. All the structures were solved by direct methods using the SIR92 program[2] and refined by full matrix least-squares on F^2 including all reflections (SHELXL97).[3] All non-hydrogen atoms were refined anisotropically. H atoms were included at calculated positions and treated as riding atoms with isotropic thermal motion related to that of its parent atom. All the calculations for these structures were performed using the WINGX crystallographic software package.[4] Table S1 gathers the crystal data and structure refinement parameters. It deserves to note that PLATON[5] Addsym routine detects a possible pseudo-translation (c/2) at 0 -0.004 1/2, but the nonfits (ca. 8%) include C5 atom of the imidazolic ring, which implies to rule out the proposition. In fact, its consideration leads to persistently high R1 values (12-13%) and the resulting structure require modelling a disorder of imidazolium cation in two positions. Furthermore, the thermal parameters of the disordered atoms are not clearly defined and require hard restrains to make the refinement stable. All in all, proposed pseudo-translation has been disregarded, in such a way that the herein reported structure is featured by lacking of any disordered entity and by exhbiting reasonable R-factors (R1 = 5.3%) and anisotropic thermal displacement parameters.

Variable temperature synchrotron X-Ray powder data collection:

Synchrotron X-ray powder diffraction (SR-XRPD) measurements were performed at the high resolution station of the MSPD beamline (BL04) at ALBA synchrotron.[6] The sample was introduced into a 0.7 mm glass capillary and measured in transmission at an energy of 20 keV (0.61872 Å wavelength determined from a Si NIST-640d reference) using the microstrip Mythen-II detector (six modules, 1280 channels/module, 50 μm/channel, sample-to-detector distance 550 mm). Temperature was controlled using an Oxford Cryosystems Series 700 Cryostream. Data from 2 to 45º (2θ) were collected during a 5 K/min ramp from 400 to 100 K every 30 s. The crystal structures were solved by the direct-space methodology implemented in TALP[6] software using intensities extracted with DAJUST[7] program. The final restrained Rietveld refinement was performed with the computer RIBOLS18[8] program.

Electrochemical Impedance Spectroscopy (EIS):

In order to study the ionic conductivity of the material, a 0.53 x 0.27 cm pellet was prepared. This pellet was not sintered since the compound decomposes at high temperature (ca. 130°C). The electrical properties were determined for the plane-parallel sample, performing ac complex impedance measurements with a Solartron 1260 Impedance Analyzer. The measured frequency range was 10^{-2}-10^{6} Hz, with 10 mV signal amplitude. The behavior of the material was studied between room temperature (R.T.) and 400 K (above this temperature, the material becomes corrosive and damages the current collectors). The impedance diagrams were analyzed and fitted by the software Zview.

Magnetization measurements

DC magnetic susceptibility measurements were performed using a Quantum Design PPMS magnetometer whilst heating from 2 to 300 K under an applied magnetic field of strength at 1 and 10 kOe. Magnetization as a function of field (H) was measured using the same magnetometer in the $-50 \leq H/kOe \leq 50$ at 2 K after cooling the sample in zero field.

Neutron diffraction experiments:

Neutron powder diffraction measurements were performed on D2B and D1B powder diffractometers at the Institute Laue-Langevin (ILL, Grenoble, France). *Ca.* 2 g of (trimim)[$FeCl_4$] were used for the experiments, which were placed in a cylindrical vanadium container and held in a liquid helium cryostat. The high-resolution powder diffractometer D2B (λ=1.5942 Å) was used to obtain extensive and accurate structural data at 250 K over a large angular angle $5 \leq 2\theta \leq 160^{o}$. High flux and medium resolution diffractometer D1B, operated at λ = 2.525 Å, was used to study the evolution of the sample in the temperature range 10–250 K. Besides, neutron diffraction patterns were measured at 250, 10 and 1.5 K in the angular range $5 \leq 2\theta \leq 128^{o}$. The collected data allowed us to solve and refine the magnetic structure.

1. Characterization of (trimim)I and (trimim)Cl

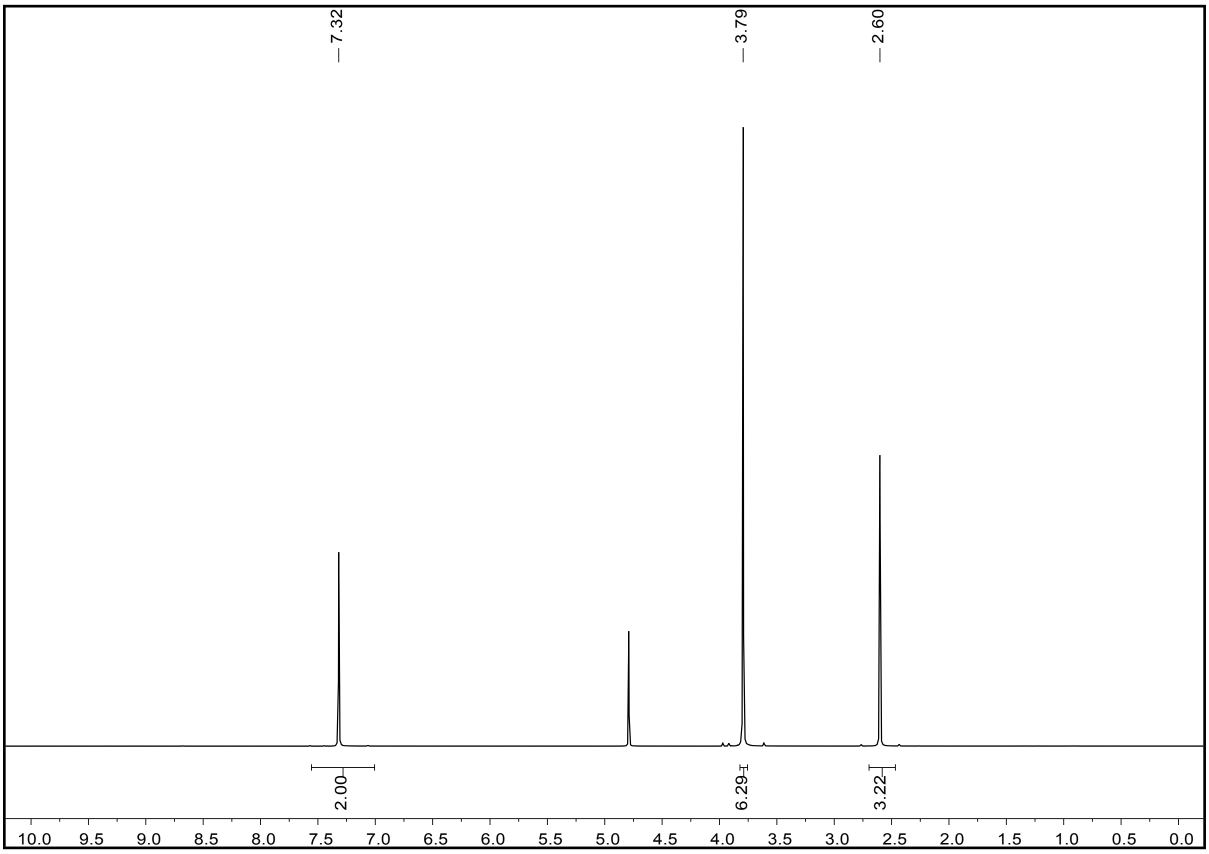

Fig. S1. ^{1}H NMR spectrum of (trimim)I (500 MHz, 298 K, D_2O).

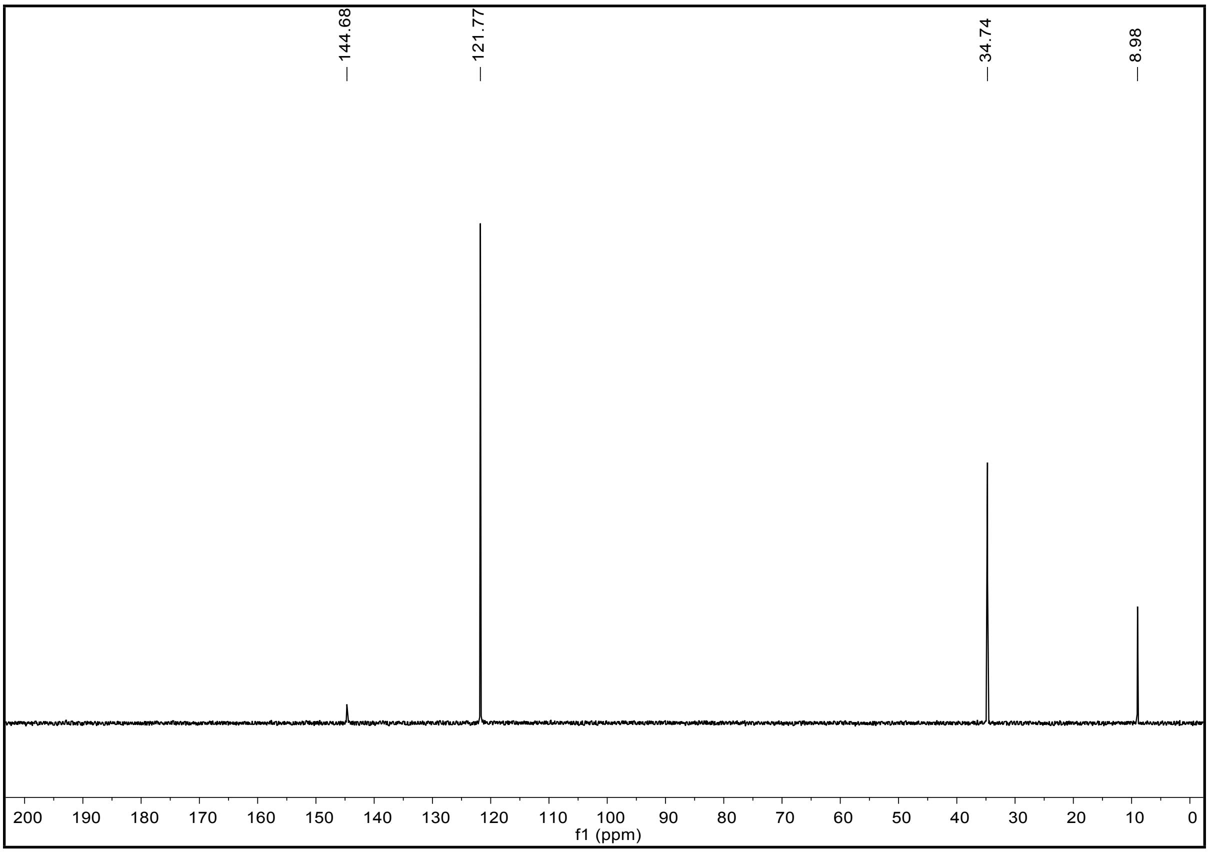

Fig. S2. $^{13}C\{^{1}H\}$ NMR spectrum of (trimim)I (126 MHz, 298 K, D_2O).

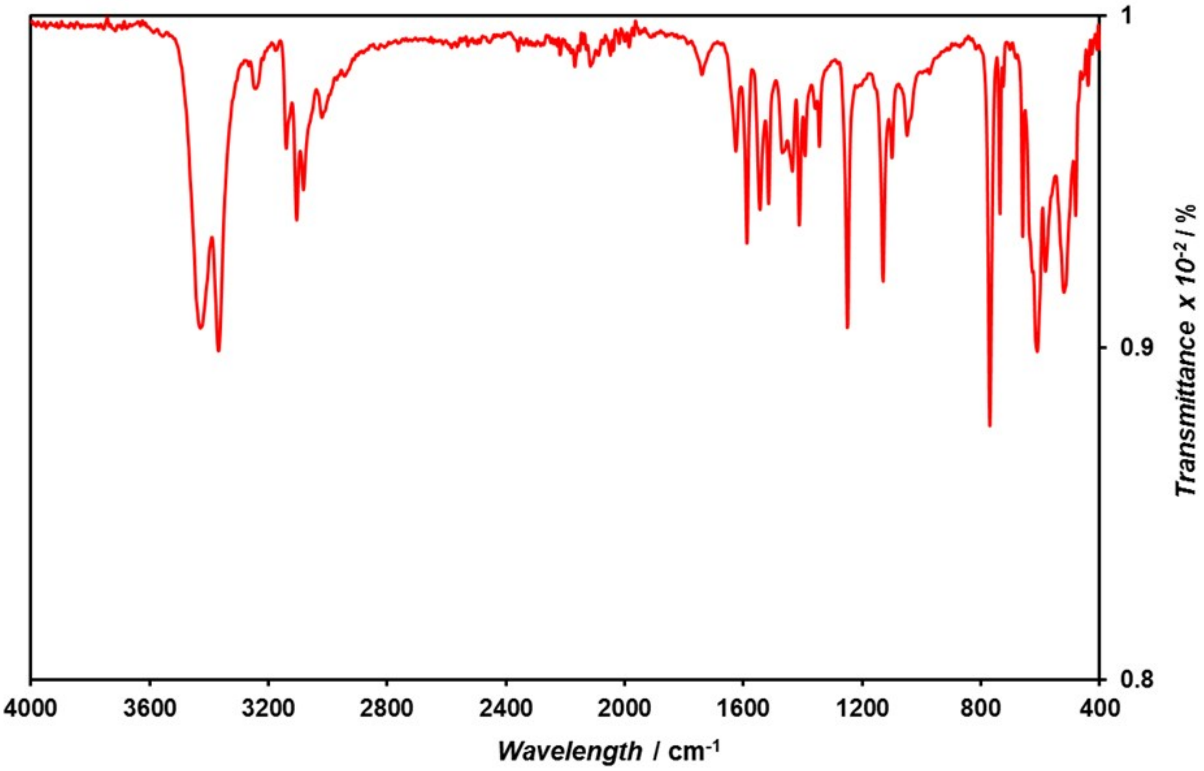

Fig. S3. IR spectrum of (trimim)I.

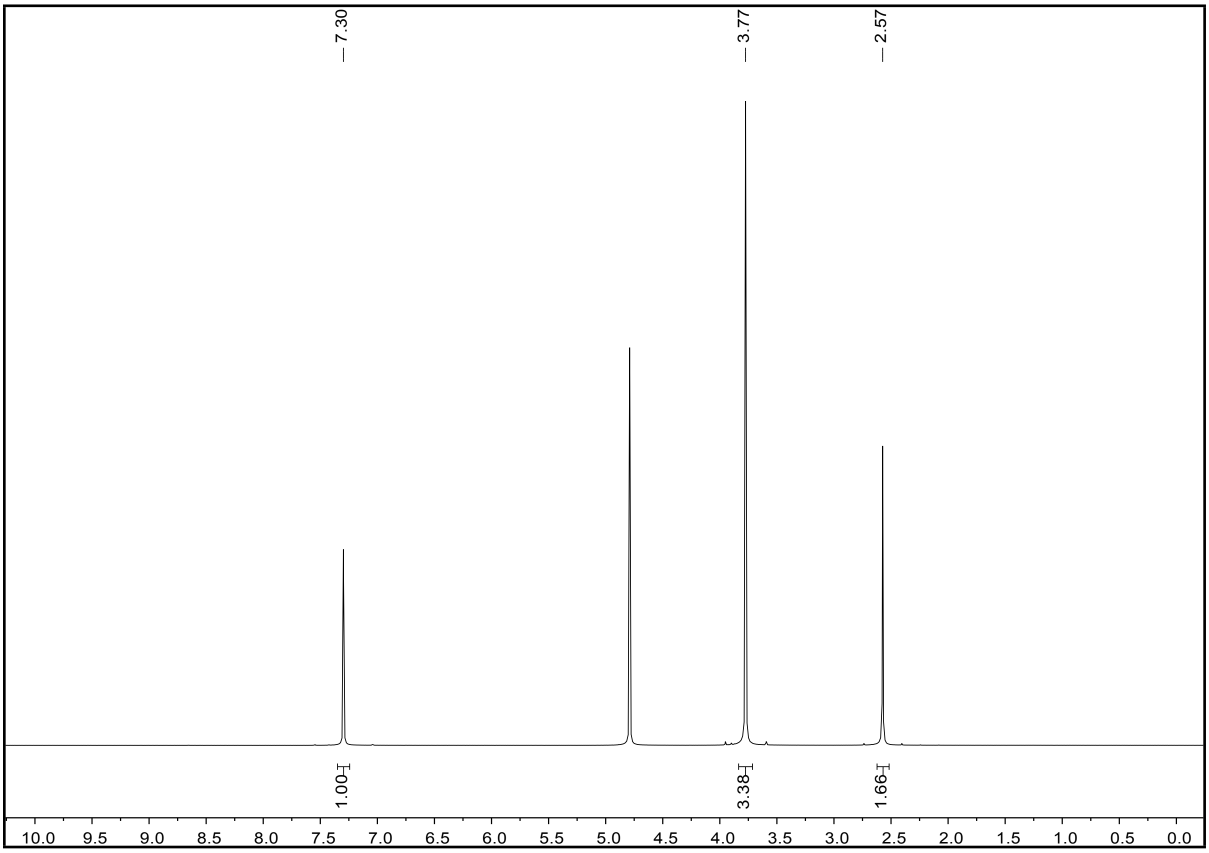

Fig. S4. ^{1}H NMR spectrum of (trimim)Cl (500 MHz, 298 K, D_2O).

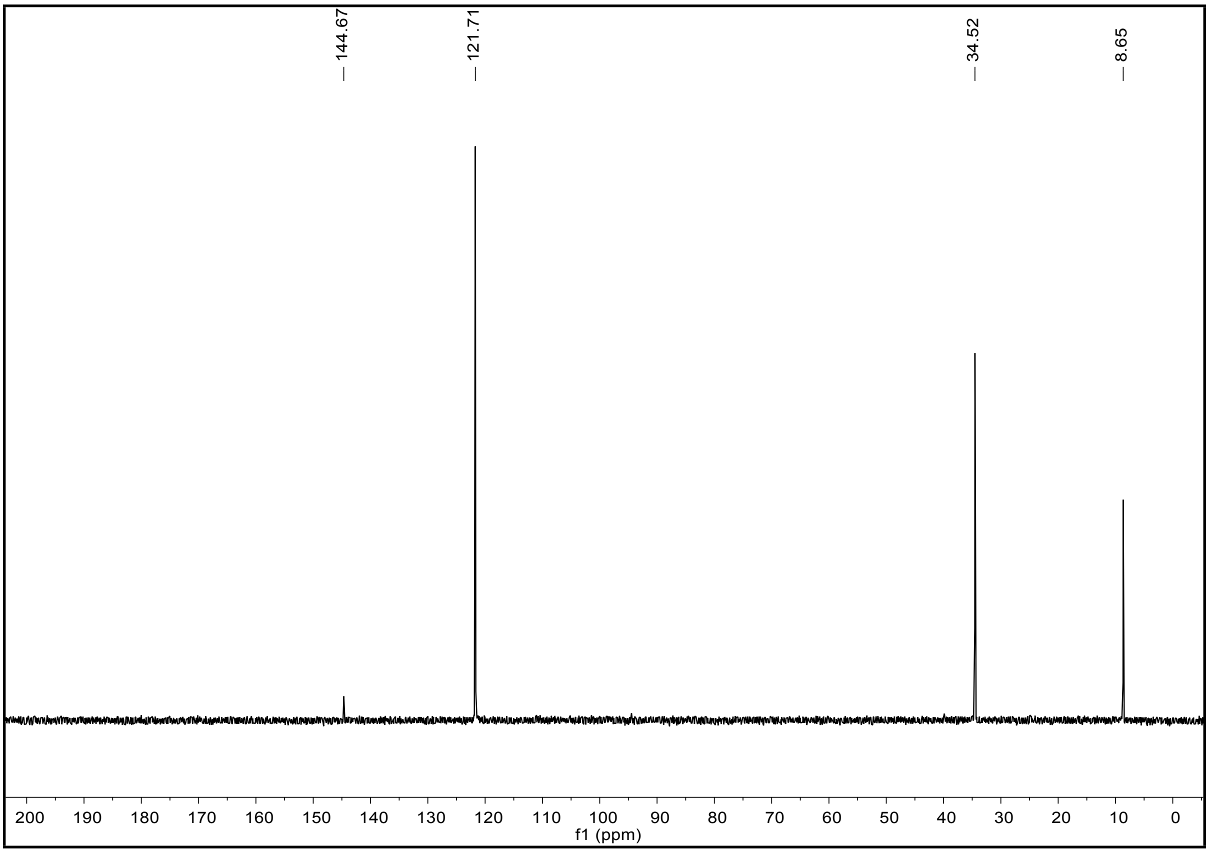

Fig. S5. $^{13}C\{^1H\}$ NMR spectrum of (trimim)I (126 MHz, 298 K, D_2O).

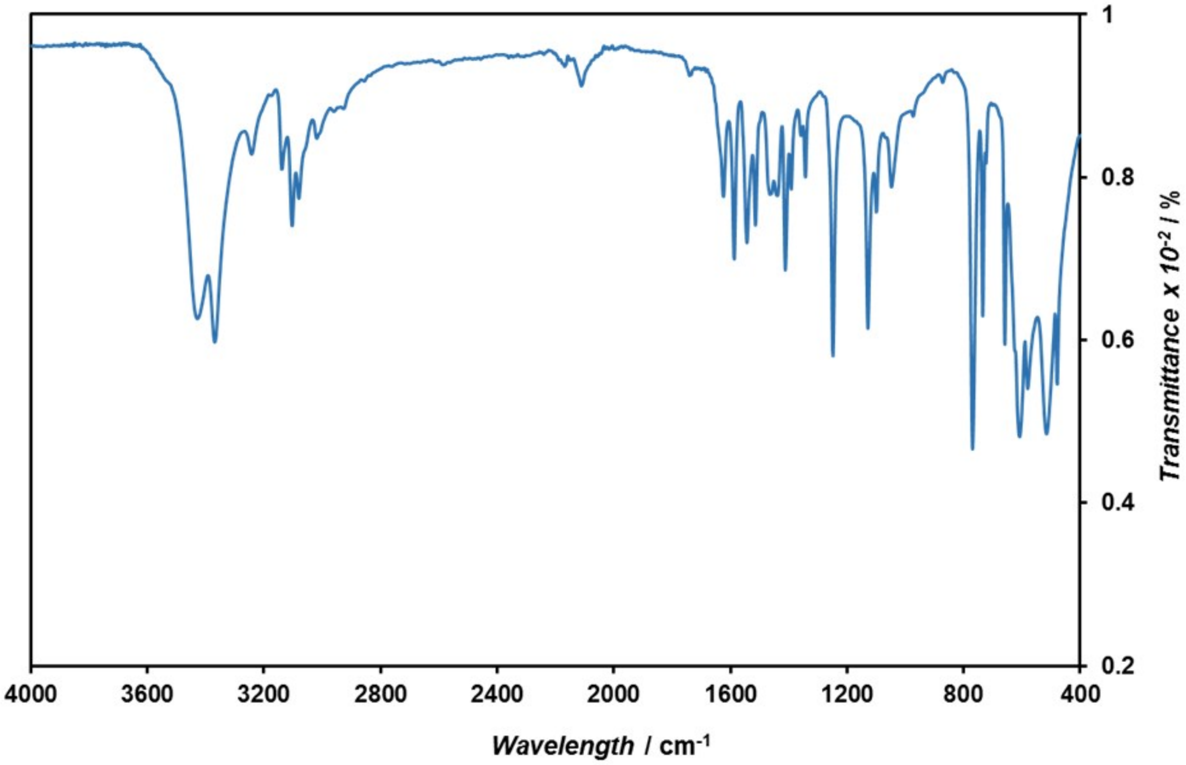

Fig. S6. IR spectrum of (trimim)Cl.

2. DSC analysis

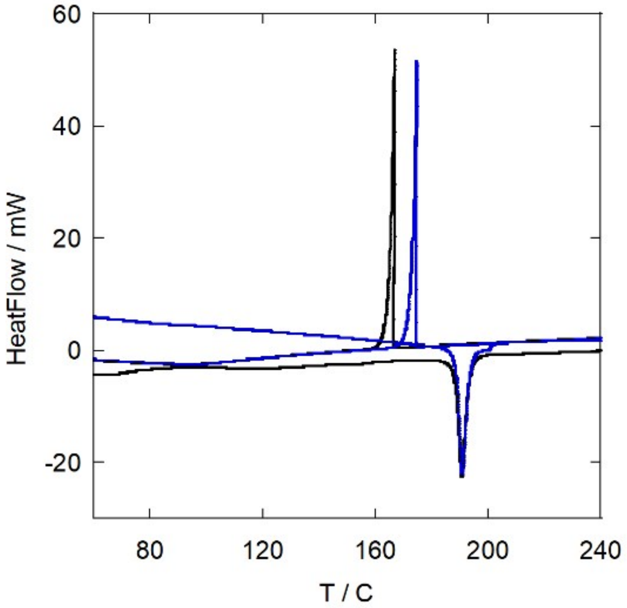

Fig. S7. DSC-thermogram of (trimim)[$FeCl_4$]. First and second cycles represented in black and blue, respectively. Heating rate: 10 K/min.

3. Variable Temperature NPD

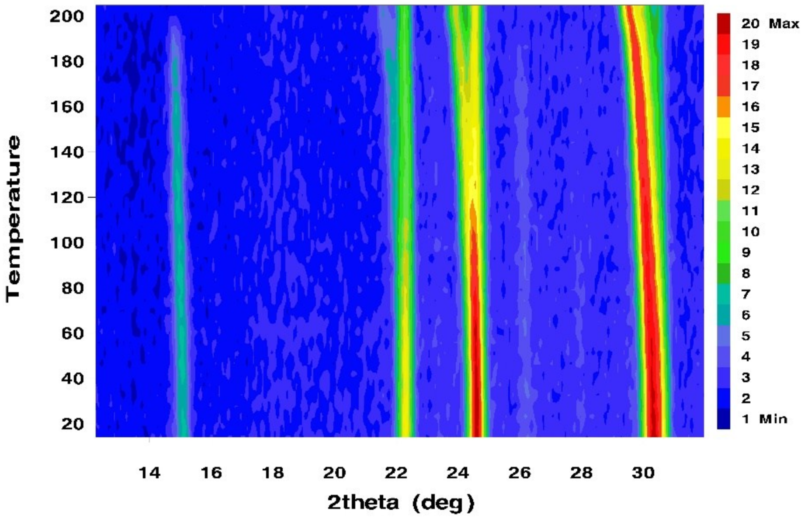

Fig. S8. NPD thermodiffractogram of (trimim)[$FeCl_4$] acquired at D1B from 10 to 220 K. The disappearance of the first Bragg peak indicates the occurrence of a nuclear phase transition at 200 K.

4. SXPD and NPD (D2B) diffraction analysis

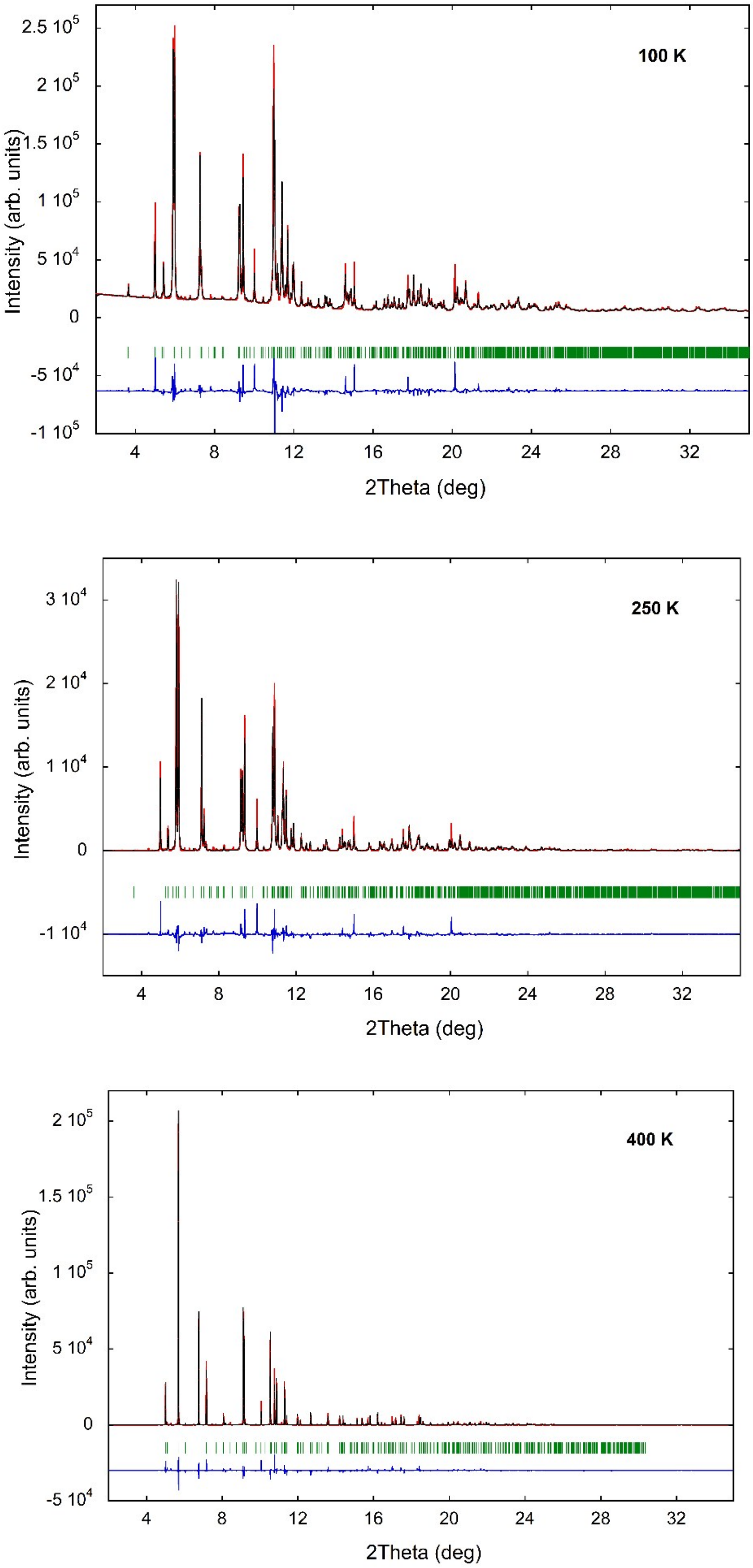

Fig. S9. Rietveld refinement of the powder diffraction pattern collected at MSPD from ALBA synchrotron at 100, 250 and 400 K. Observed (red points) and calculated (black solid line) powder diffraction patterns

for positions of the Bragg reflections are represented by green vertical bars. The observed-calculated difference patterns are depicted as a blue line.

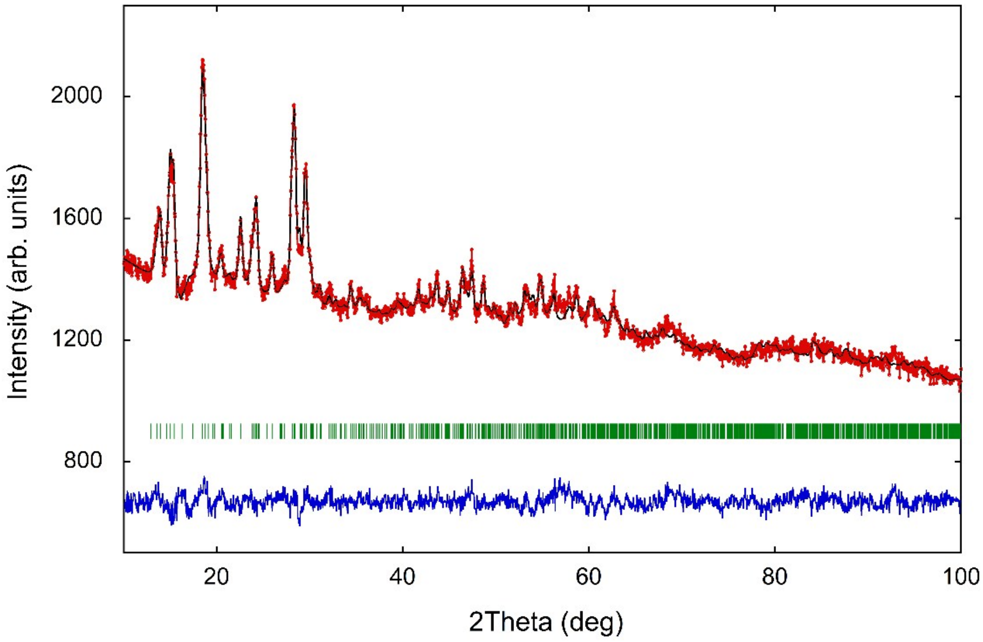

Fig. S10. Rietveld refinement of the powder diffraction pattern collected at D2B at 250 K. Observed (red points) and calculated (black solid line) neutron diffraction intensity. Positions of the Bragg reflections are represented by green vertical bars. The observed-calculated difference patterns are depicted as a blue line. The R_{Bragg} after last refinement cycle was of 11.7%.

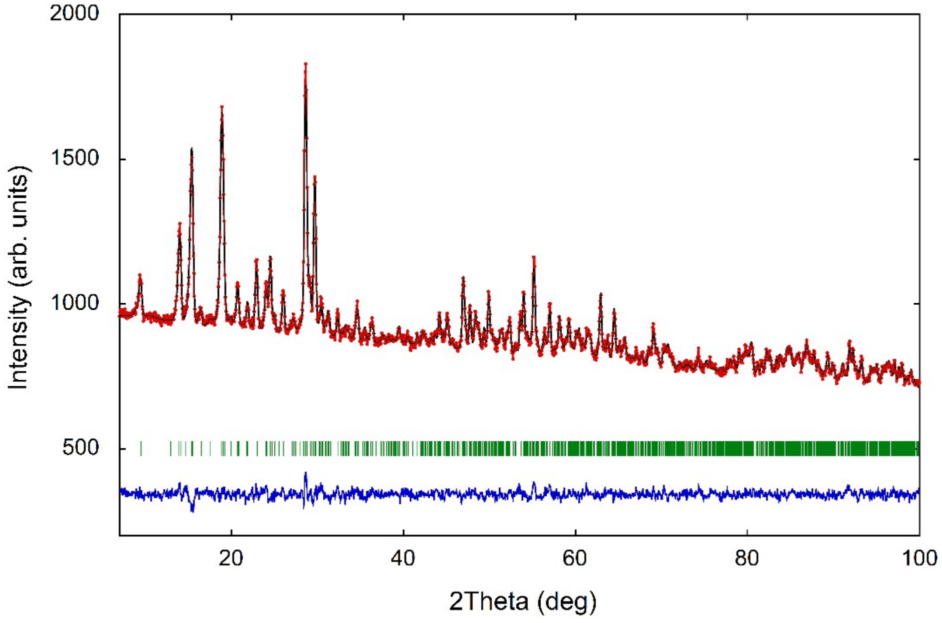

Fig. S11. Rietveld refinement of the powder diffraction pattern collected at D2B at 100 K. Observed (red points) and calculated (black solid line) neutron diffraction intensity. Positions of the Bragg reflections are represented by green vertical bars. The observed-calculated difference patterns are depicted as a blue line. The R_{Bragg} after last refinement cycle was of 6.85%.

5. Ionic conductivity as a function of temperature

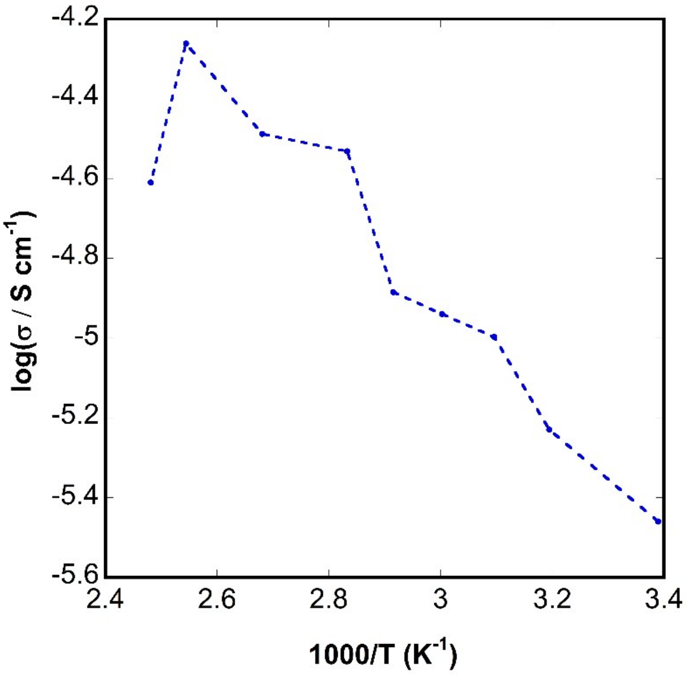

Fig. S12. Ionic conductivity as a function of temperature obtained from the fit to the impedance spectra.

6. Curie-Weiss fitting

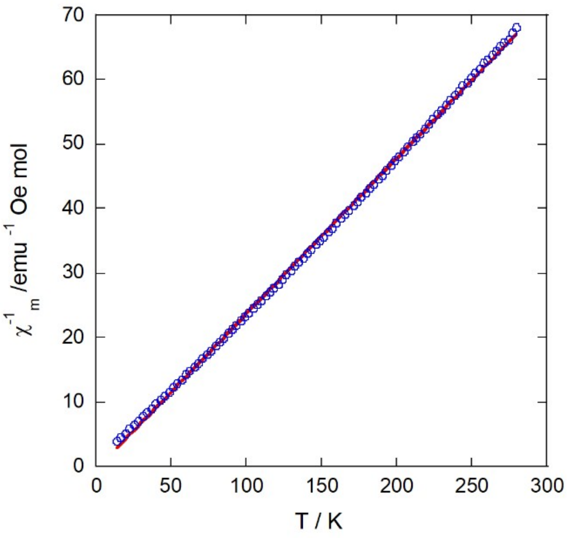

Fig. S13. Fit to the Curie-Weiss law (solid red line) to the magnetic susceptibility data at 10 kOe.

7. Tables

Table S1. Melting temperatures of different imidazolium-based haloferrate compounds.

	T_m (C)	
	$FeCl_4$	$FeBr_4$
**emim**$^{+}$ [7]	12	
**edimim**$^{+}$ [8]	47	82
**dimim**$^{+}$ [9,10]	91	96
**trimim**$^{+}$	192	210

Table S2. Crystallographic data and single-crystal refinement details of (trimim)[$FeCl_4$] at 150 K (i.e. II_b).

	(trimim)[$FeCl_4$]
Empirical formula	$C_6H_{11}Cl_4FeN_2$
Formula weight	308.82
Crystal system	monoclinic
Space group	$P2_1/c$
Temperature (K)	150
a (Å)	6.541(5)
b (Å)	14.158(5)
c (Å)	13.351(5)
β (º)	95.590(5)
V (Å^3)	1230.5(11)
Z	4
ρ (g·cm^{-3})	1.667
μ (mm^{-1})	2.053
Reflections collected	8114
Unique data/parameters	2542/121
R	0.0484
Goodness of fit (S)[a]	1.158
R_1[b]/wR2[c] [I>2σ(I)]	0.0528/0.1365
R_1[b]/wR2[c] [all data]	0.0658/0.1445

[a] $S = [\sum w(F_0^2 - F_c^2)^2 / (N_{obs} - N_{param})]^{1/2}$. [b] $R_1 = \sum||F_0|-|F_c|| / \sum|F_0|$; [c] $wR2 = [\sum w(F_0^2)^2 - F_c^2)^2 / \sum w(F_0^2)^2]^{1/2}$; $w = 1/[\sigma^2(F_0^2) + (aP)^2 + bP]$ where $P = (max(F_0^2,0) + 2Fc^2)/3$, being a = 0.0528 and b = 3.3176

Table S3. Capacitance and resistance values obtained from the impedance spectra at different temperatures.

	**High Frequencies**		**Low Frequencies**	
**Temperature (ºC)**	**C (F)**	**R (ohm)**	**C (F)**	**R (ohm)**
130	$1.955\cdot10^{-11}$	12459	$1.395\cdot10^{-7}$	3192
120	$1.881\cdot10^{-11}$	5587	$1.545\cdot10^{-6}$	963
100	$1.949\cdot10^{-11}$	9414	$3.245\cdot10^{-6}$	725
80	$1.967\cdot10^{-11}$	10407		
70	$1.915\cdot10^{-11}$	23471		
60	$1.903\cdot10^{-11}$	26631		
50	$1.924\cdot10^{-11}$	30401		
40	$1.883\cdot10^{-11}$	51942		
22	$1.848\cdot10^{-11}$	88261		

8. Details of magnetic model calculations

The total magnetic representation of the propagation vector group [$\boldsymbol{k}$ = (0, 0, 0)] can be decomposed on four irreducible representations, each one with three basis vectors. Therefore, the Γ_{mag} can be represented as:

$\Gamma_{\text{Magnetic}} = 3\Gamma(1) \oplus 3\Gamma(2) \oplus 3\Gamma(3) \oplus 3\Gamma(4)$

The different magnetic structures can be deduced from the Fourier coefficient shown in Table S1.

Table S6. Expressions of the Fourier coefficients, obtained for the Fe^{3+} site, for the $P\ 2_1/c$ space group and (0, 0, 0) propagation vector.

	x,y,z	-*x*,*y*+1/2,-*z*+1/2	-*x*,-*y*,-*z*	*x*,-*y*+1/2,*z*+1/2	S.G.
Γ(1)	(*u*, *v*, *w*)	(*u*,-*v*,*w*)	(*u*, *v*, *w*)	(*u*,-*v*,*w*)	$P\ 2_1/c$
Γ(2)	(*u*, *v*, *w*)	(-*u*,*v*,-*w*)	(-*u*,-*v*,-*w*)	(*u*,-*v*,*w*)	$P\ 2_1/c'$
Γ(3)	(*u*, *v*, *w*)	(*u*,-*v*,*w*)	(-*u*,-*v*,-*w*)	(-*u*,*v*,-*w*)	$P\ 2_1'/c'$
Γ(4)	(*u*, *v*, *w*)	(-*u*,*v*,-*w*)	(*u*,*v*,*w*)	(-*u*,*v*,-*w*)	$P\ 2_1'/c$

As shown in table S6, there are four potential maximal magnetic space groups, corresponding to $P\ 2_1/c$, $P\ 2_1/c'$, $P\ 2_1'/c'$ and $P\ 2_1'/c$. The magnetic space groups $P\ 2_1/c$ and $P\ 2_1'/c'$ can be excluded, as they lead to a neat component of the magnetic moment (ferrimagnetic) which is not compatible with the magnetometric measurements. From this analysis, there are only two remaining possibilities. In the $P\ 2_1'/c$ space group, the (010) reflexion, which is the most intense, is not signaled and thus this magnetic space group can also be excluded. Consequently, the only possible solution is the magnetic space group $P\ 2_1/c'$. The fit of the data to this space group can be found in the manuscript.

9. References

1. Agilent. *CrysAlis PRO* Agilent Technologies Ltd, Yarnton, England, 2010.

2. A. Altomare, G. Cascarano, C, Giacovazzo and A. Guagliardi, *J. Appl. Crys.* 1993, **26**, 343-350.

3. G. M. Sheldrick, *Acta Crys.* 2008, **A64**, 112-122.

4. L. J. Farrugia, *J. Appl.Cryst.* 1999, **32**, 837-838.

5. A. L. Spek, *Acta Cryst.* 2009, **D65**, 148-155.

6. O. Vallcorba, J. Rius, C. Frontera and C. Miravitlles, *J. Appl. Crystallogr.* 2012, **45**, 1270-1277.

7. O. Vallcorba, J. Rius, C. Frontera, I. Peral and C. Miravitlles, *J. Appl. Crystallogr.* 2012, **45**, 844-848.

8. J. Rius, *RIBOLS18- A computer program for least-squares refinement from poder diffraction data; Institut de Ciència de Materials de Barcelona (CSIC): Barcelona, Spain.* 2012.

9. F. Fauth, I. Peral, C. Popescu and M. Knapp, *Powder Diffraction.* 2013, **28-S2**, S360-S370.

7 A. García-Saiz, I. de Pedro, J. A. Blanco, J. González and J. Rodríguez Fernández, *The J.Phys.Chem. B,* 2013**, 117**, 3198–3206.

8 A. García-Saiz, I. de Pedro, O. Vallcorba, P. Migowski, I. Hernández, L. Fernández Barquin, I. Abrahams, M. Motevalli, J. Dupont, J. A. Gonzalez and J. Rodríguez Fernández, *RSC Adv*., 2015, **5**, 60835-60848.

9 A. García‐Saiz, P. Migowski, O. Vallcorba, J. Junquera, J. A. Blanco, J. A. González, M. T. Fernández‐Díaz, J. Rius, J.; Dupont and J. Rodríguez Fernández *Chem. Eur. J,.* 2014, **20**, 72–76.

10 A. García-Saiz, I. de Pedro, P. Migowski, O. Vallcorba, J. Junquera, J. A. Blanco, O. Fabelo, D. Sheptyakov, J. C. Waerenborgh, M. T. Fernández-Díaz, J. Rius, J. Dupont, J. A. Gonzalez and J. Rodríguez Fernández, *Inorg. Chem*., 2014, **53**, 8384–8396.
